# Numerical Modeling of the Mixing of Highly Viscous Polymer Suspensions in Partially Filled Sigma Blade Mixers

**DOI:** 10.3390/polym15081938

**Published:** 2023-04-19

**Authors:** Michael Roland Larsen, Tobias Ottsen, Erik Tomas Holmen Olofsson, Jon Spangenberg

**Affiliations:** 1 Department of Mechanical Engineering, Technical University of Denmark, 2800 Kgs. Lyngby, Denmark; etho@mek.dtu.dk; 2 Dansac A/S, 3480 Fredensborg, Denmark; 3 Manex ApS, 3500 Værløse, Denmark; 4 Haldor Topsoe A/S, 2800 Kgs. Lyngby, Denmark

**Keywords:** mixing, suspensions, homogeneity, computational fluid dynamics, viscous dissipation, distributive- and dispersive-mixing

## Abstract

This paper presents a non-isothermal, non-Newtonian Computational Fluid Dynamics (CFD) model for the mixing of a highly viscous polymer suspension in a partially filled sigma blade mixer. The model accounts for viscous heating and the free surface of the suspension. The rheological model is found by calibration with experimental temperature measurements. Subsequently, the model is exploited to study the effect of applying heat both before and during mixing on the suspension’s mixing quality. Two mixing indexes are used to evaluate the mixing condition, namely, the Ica Manas-Zlaczower dispersive index and Kramer’s distributive index. Some fluctuations are observed in the predictions of the dispersive mixing index, which could be associated with the free surface of the suspension, thus indicating that this index might not be ideal for partially filled mixers. The Kramer index results are stable and indicate that the particles in the suspension can be well distributed. Interestingly, the results highlight that the speed at which the suspension becomes well distributed is almost independent of applying heat both before and during the process.

## 1. Introduction

Attaining and ensuring homogeneity in particle-based slurries is not a trivial task—not least due to the complexity in quantifying homogeneity. The problem only gets more complicated when dealing with highly viscous non-Newtonian fluids. However, applications where homogeneity control is paramount are broad, extending to many industries such as food, pharmaceuticals, concrete and medical devices.

Some of the earliest attempts to obtain a quantification of homogeneity were made by Lacey in 1954 [1] and later by P. V. Danckwerts [2]. Both stated the importance of the sampling procedure, which is essential in determining whether particles in a population were evenly distributed. Other studies have used density as a method for the quantification of homogeneity [3,4]. These approaches have used standard statistics methods such as Coefficient of Variance [3] or Analysis of Variance (ANOVA) [4]. This works if you have appreciable unevenness in the distribution and a significant density discrepancy between components [5].

One of the pioneers within the field of mixing is Ica Manas-Zlaczower [6,7,8,9,10,11]. Her famous dispersive mixing index from 1992 is still used today [12,13,14,15,16,17], along with her distributive Cluster Distribute Index [12,17,18]. There have not been many alternatives to Zlaczower’s dispersive mixing index. On the other hand, there are a number of alternatives to the Cluster Distribution Index—for example, the Scale of Segregation, a distributive mixing index that was developed back in 1952 [19] and later applied in Computational Fluid Dynamics (CFD) models by Connelly and Kokini in 2007 [20]. Lastly, there is the Lacey mixing index [21], which has been modified over the years. Other indices that have been developed based on the Lacey mixing index include the Kramer mixing index [22] and the Ashton–Valentin mixing index [23].

Heating is an important aspect of mixing, since it can reduce viscosity drastically [12,17,24,25]. The advantage of reducing the viscosity is that it leads to an increased Reynolds Number, which improves mass transfer (i.e., mixing) [25]. The viscous dissipation of heat is a phenomenon that is often neglected in fluid dynamics [25]. However, when dealing with highly viscous fluids, viscous heating can be present if they are mixed with a certain force. Consequently, this leads to an impact on the energy balance and should therefore only cautiously be ignored, as shown in previous studies [12,26,27].

Sigma blades can often be used as the rotating mixing element in a mixer when dealing with highly viscous fluids [28]. They can be used either as a single-element [3] or, as often seen, as a twin-arm mixer [29]. Sigma blades have previously been simulated by Connelly [30,31] and evaluated with respect to the dispersive mixing index. However, to the best knowledge of the authors, no numerical models have been presented in the literature that account for a free surface, a non-Newtonian material behavior and viscous dissipation when mixing with Sigma blades.

This paper presents a new non-isothermal, non-Newtonian CFD model for the sigma blade mixing of an adhesive suspension, where both the free surface and viscous heating are accounted for. The suspension is modeled as a viscoplastic fluid, and model calibration is performed via optical temperature measurements. To evaluate the mixing quality, we have used Zlaczower’s dispersive mixing index as well as Kramer’s distributive mixing index. The model is used to investigate the effect of applying heat both before and during the process on the mixing quality. The rest of the paper is organized as follows: Section 2 introduces the experimental setup as well as the numerical model, while in Section 3, the results are presented and discussed. Section 4 summarizes the conclusions of the study.

## 2. Materials and Methods

### 2.1. Experiments

The mixing material consists of a highly viscous polymer fluid and a multi-component powder blend. The powder contains both colloidal and microscale particles. The specific fluid/powder suspension is a piece of intellectual property and cannot be disclosed. The viscosity of suspensions changes with the volume fraction of the powder [32,33], but this is not accounted for in the numerical model, as it is assumed to have a limited effect on the results. The physical data, besides viscosity, are shown in Table 1. The density measurements were performed with a Sartorius YDK03 Density Kit for Analytical Balances, which uses the Archimedes principle. The heat capacity and conductivity measurements were performed on a TCi-3-A from C-Therm Technologies Ltd., which uses the Modified Transient Plane Source (MTPS) method that has been used in textile research [34].

**Figure 1:**
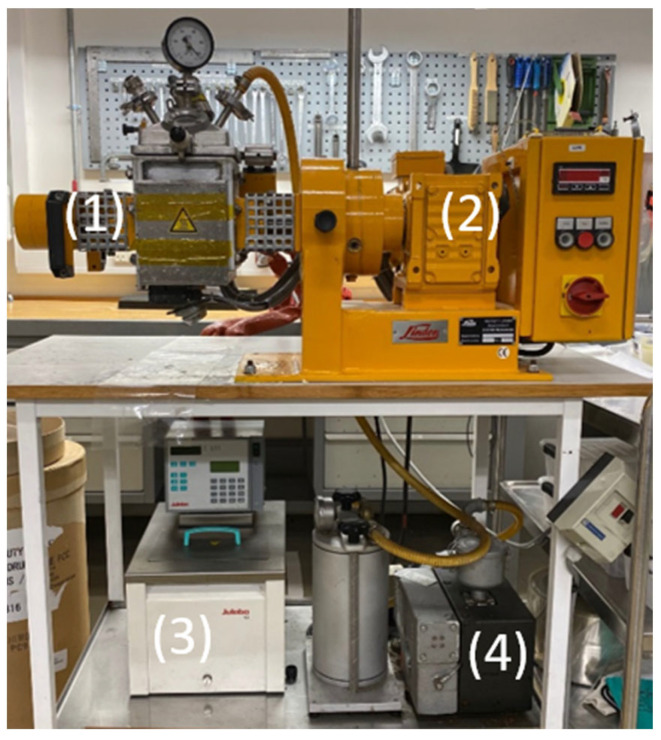
The mixing setup. (1) Chamber of the mixer, (2) control panel and digital display of the temperature, (3) heat exchanger, (4) vacuum pump.

The experimental mixing was carried out on a Linden LK II 1 machine; see Figure 1. The machine has a mixing chamber with two sigma blades rotating in opposite directions. The front blade rotates at 60 rpm, and the one behind rotates at 19 rpm. Historically, this has been a typical setting for highly viscous mixing [28]. The mixer also has a control panel to switch the rotation direction. In addition, there is a digital display that indicates the temperature, as measured at the bottom of the mixer. Attached to the mixing equipment is a heat exchanger that adjusts the temperature for the mixing process. A vacuum pump is also mounted to ensure that air is not trapped inside the mixture. Two walls in the mixer can provide heat to the system. If this function is on, the temperature will be 80 °C.

### 2.2. Numerical Model

**Figure 2:**
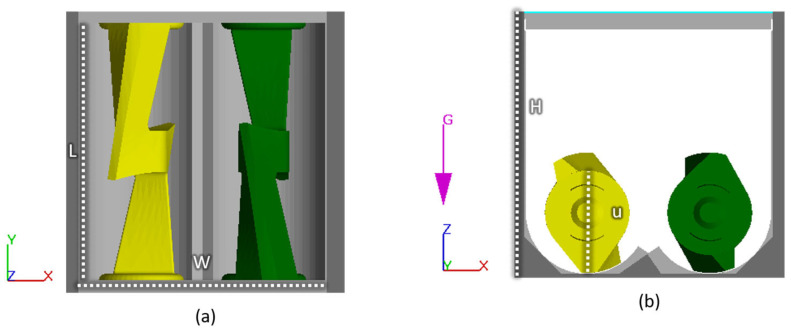
Illustration of the mixer geometry from (**a**) above and (**b**) the side. W and H have a distance of 120 mm, while L and u are 126.4 mm and 50.23 mm, respectively.

The CFD model simulates the mixing of the suspension in the mixing chamber and is developed in the commercial software FLOW-3D, which has successfully simulated other processes such as casting [35,36] and 3D printing [37,38] highly viscous fluids. An illustration of the mixer geometry is seen in Figure 2. At t=0, the fluid is well distributed at the bottom, and the powder is placed on top of the fluid. At t>0, the sigma blades start rotating. A no-slip boundary condition is applied on all solid surfaces. The computational domain is meshed with a uniform grid consisting of ~50,000 elements, which was arrived at after a mesh sensitivity analysis. The two side walls perpendicular to the sigma blades apply heat to the system; see Figure 2.

The flow is transient and non-isothermal since the viscosity is temperature dependent. The material is modeled as an incompressible substance, and thus, the density is approximated as constant. Hence, the flow is computed by considering the mass, momentum and energy conservation:

(1) $$\frac{\partial \rho }{\partial t}+\left(\nabla \cdot \rho v\right)=0$$

(2) $$\rho \frac{Dv}{Dt}=-\nabla p-\left[\nabla \cdot \tau \right]+\rho G$$

(3) $$\rho C_{p}\frac{DT}{Dt}=-\left(\nabla \cdot q\right)-\left(\tau :\nabla v\right)$$

(4) q=−k∇T

where ρ is the density, p is the pressure, k is the thermal conductivity $C_{p}$, is the specific heat capacity and q is the heat flux vector. τ is the material deviatoric stress tensor and is calculated as $\tau =2\mu \left(\dot{\gamma },T\right)D$. D is the trace of the deformation rate tensor, and it is defined as $D=\frac{1}{2}\left(\nabla v\right)+\nabla v^{T}$. $\dot{\gamma }$ is the shear rate and is calculated by $\dot{\gamma }=\sqrt{2\mathrm{tr}\left(D^{2}\right)}$. The gravitational acceleration, G, is given by $\left(\left(0,0,-9.82\right)\frac{m}{s^{2}}\right)$. The software used the finite volume method to discretize the governing equations. The equation of energy is calculated explicitly, while the viscous stress and pressure are solved implicitly. The advection is solved explicitly with first-order accuracy. The free surface is calculated with the volume of fluid technique [39].

**Figure 3:**
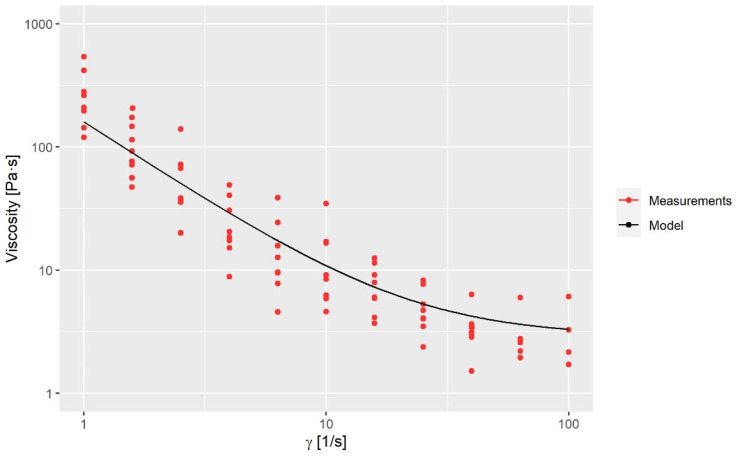
Viscosity measurements compared to the model.

The material behaves as a non-isothermal viscoplastic fluid and is simulated by a modified Carreu model:

(5) $$\mu \left(\dot{\gamma },T\right)=\left\{\begin{array}{c} \mu_{max}\;\begin{array}{ccc} , & \;\;\;for & \dot{\gamma }\leq {\dot{\gamma }}_{c} \end{array}\\ \begin{array}{ccc} \mu_{\infty }+\frac{\mu_{0}E\left(T\right)-\mu_{\infty }}{{\left({\dot{\gamma }}^{2}{\left(\lambda E\left(T\right)\right)}^{2}\right)}^{\frac{1-n}{2}}}, & for & \dot{\gamma }>{\dot{\gamma }}_{c} \end{array} \end{array}\right.$$

(6) $$E\left(T\right)=\exp\left(a\left(\frac{T^{ref}}{T-b}\right)\right)$$

where $\mu_{0}$ is the zero-share-rate viscosity, $\mu_{\infty }$ is the infinity-share-rate viscosity, n is the power exponent, λ is the time constant, E is an energy function which is dependent on the fluid temperature, a and b are empirical constants and $T^{ref}$ is the reference temperature. The applied values of the variables in Equations (5) and (6) are seen in Table 2. The shear rate-dependent variables are obtained via a rheological characterization of the fluid; see Figure 3. The temperature-dependent variables are obtained by a calibration, as the fluid was too viscous to perform measurements at low temperatures. The calibration is reported in Section 3.1.

The evaluation of the mixing is carried out by a dispersive- and distributive-mixing index. The dispersive mixing is quantified through the Manas–Zlacower mixing index, $\lambda_{mz}$; see Equation (8). ω is the vorticity. When $\lambda_{mz}$ is close to 0, the mixing is purely rotational driven, while at 0.5 and 1, the mixing is shear-driven and purely elongation-driven, respectively. The latter leads to a desirable faster breakup of agglomerates [11,13,29].

(7) $$\lambda_{mz}=\frac{\left|\dot{\gamma }\right|}{\left|\dot{\gamma }\right|+\left|\omega \right|}$$

The second approach is a distributive mixing index where an artificial concentration or material is used. The concentration is defined as a scalar with zero diffusion, which does not affect the physics (such as the viscosity and density). Thus, the only effect is pure mixing. The mean of the concentration, $\bar{c}$, is 0.5. The dimensionless concentration, $\hat{c_{i}}$, and the dimensionless variance across the domain that contains fluid, $S^{2}$, are found by Equations (8) and (9). From the variance, the Kramer dispersive mixing index, $M_{Kramer}$, can be found by Equation (10) [17], where $\sigma_{r}=\frac{\sigma_{0}}{N_{f}}$ and $\sigma_{0}={\left(P\cdot \left(1-P\right)\right)}^{\frac{1}{2}}$, with P being the proportion of the component containing the concentration, which is set to 0.5 for this paper.

(8) $$\hat{c_{i}}=\frac{c_{i}-\bar{c}}{\bar{c}}$$

(9) $$S^{2}=\frac{1}{\left(N_{f}-1\right)}\overset{N_{f}}{\underset{i=1}{\sum }}{\left(\hat{c_{i}}\right)}^{2}$$

(10) $$M_{Kramer}=\frac{\sigma_{0}-S}{\sigma_{0}-\sigma_{r}\;}$$

Table 3 presents the process parameters for the simulations that are studied in this paper.

## 3. Results and Discussion

### 3.1. Calibration

**Figure 4:**
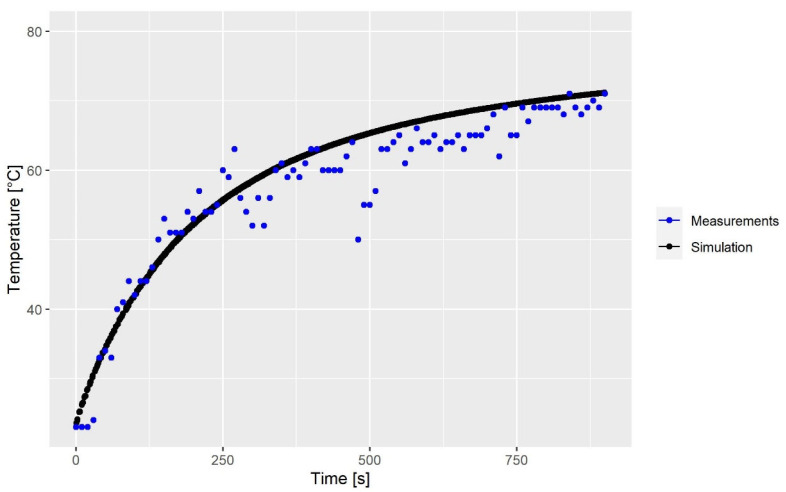
Experimental and simulation results of the surface temperature of the fluid as a function of time. The absolute mean error between the simulation and measurement is 1.72 °C.

**Figure 5:**
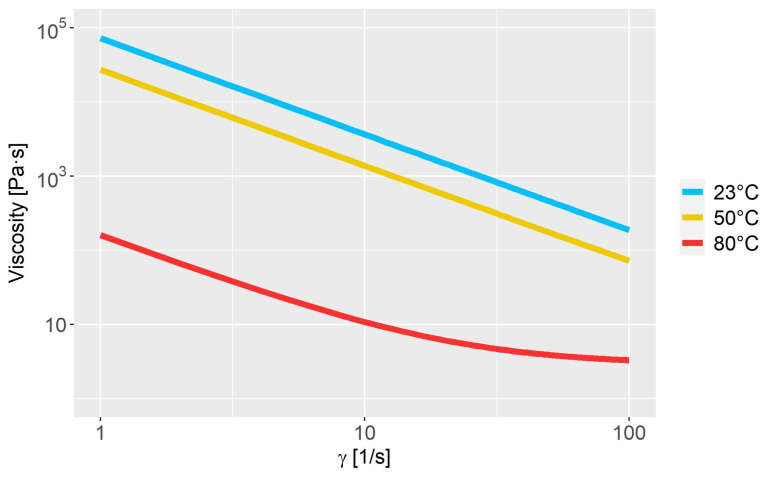
Viscosity curve as a function of the shear rate at different temperatures.

In the experimental setup, an optical thermal probe was placed inside the mixing chamber to measure the temperature of the suspension. The probe logged a surface temperature every 10 s. The initial temperature of the fluid was measured to be 23 °C, and no heat was applied while mixing. The experimental findings and the results of the calibration simulation,  CA23, are shown in Figure 4. The experimental measurements show that viscous heating is present and that the temperature increases most rapidly during the first 250 s. The fluctuation around 250 and 500 s is possibly due to the brushed steel occasionally being measured instead of the fluid. The parameters a and b in Table 2 are calibrated in order to make the CFD model predict the same temperature evolution as that seen in the experiment. By doing so, the experimental findings and the numerical results are in very good agreement. The absolute mean error between the two is 1.72 °C. The rheological model obtained by calibration is presented in Figure 5 for the temperatures 23, 50 and 80 °C.

### 3.2. Temperature Distribution

**Figure 6:**
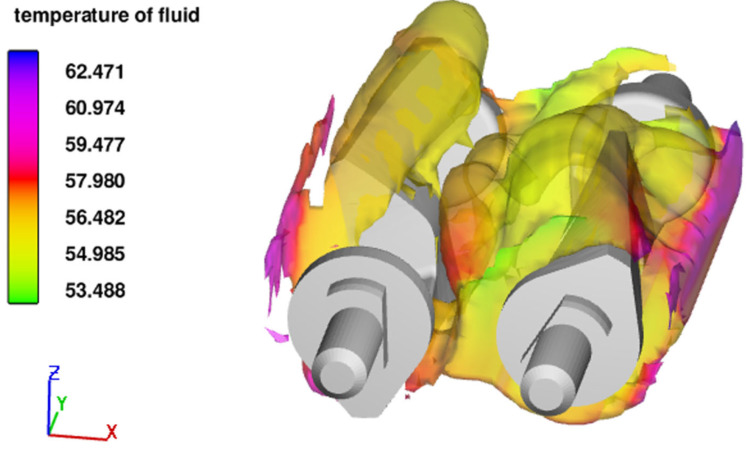
Temperature profile for NA50 at t=15  s.

The temperature distribution is close to uniform when mixed under adiabatic boundary conditions, but this is not the case when applying the non-adiabatic boundary condition, as seen in Figure 6, which illustrates the temperature profile for *NA*50 at t=15 s. The profile shows that the temperature is highest near the heated walls. The temperature decrease along the *x*-axis is due to the fluid not being exposed to the fixed wall temperature. The average temperature increases with time due to the viscous heating and heated walls.

**Figure 7:**
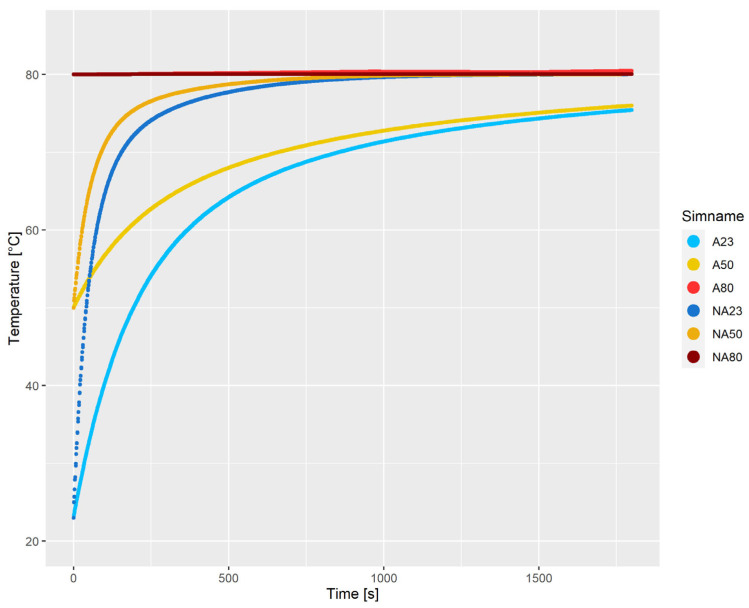
Average temperature of the fluid as a function of time.

The average temperature of the fluid as a function of time is illustrated in Figure 7 for all simulations except CA23. The simulations with applied wall temperature increase faster in terms of temperature as compared to their counterpart with adiabatic boundary conditions, except for *NA*80, where the walls effectively cool the suspension. *A*23 and *A*50 do not have enough time to reach 80 °C, while *A*80 reaches a temperature slightly higher than 80 °C.

### 3.3. Velocity Field

**Figure 8:**
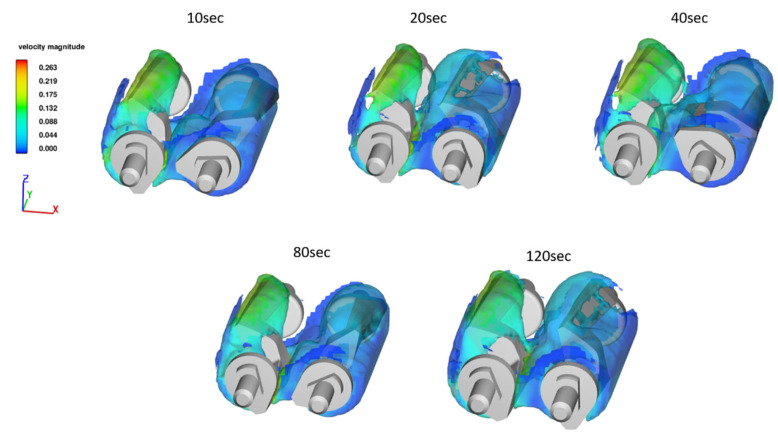
Velocity profile for an initial temperature of 80 °C with adiabatic boundary conditions at different time values. The velocity values are in m/s.

The velocity field gives a good indication of how the flow pattern is inside of the mixer. In Figure 8, the velocity field for *A*80 is presented at different times. As expected, the sigma blade at the lowest x-value is the one with the highest velocity, while the second sigma blade spins slower to ensure mixing in the whole system. Noticeably, some of the fluid sticks to the wall, which is due to the viscoplastic effect of the fluid and the no-slip condition on the walls.

### 3.4. Dispersive Mixing

**Figure 9:**
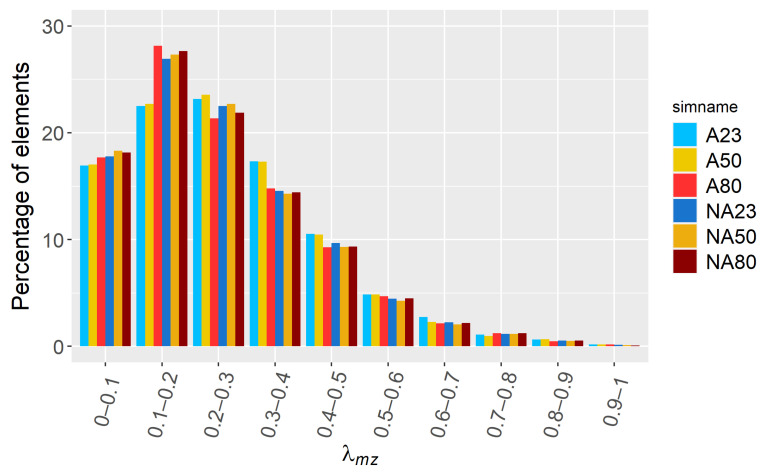
Histogram of the $\lambda_{mz}$ at t=1800 s.

In Figure 9, the histogram results of $\lambda_{mz}$ at t=1800 s are shown for all simulations except CA23. The results are fairly similar for all simulations, which indicates that preheating and heating during the process do not have a big influence on the mixing quality after 30 min. The dispersive mixing index is primarily between 0 and 0.5, which is similar to the findings of Ahmed and Chandy [12], who simulated a two-wing rotors mixer. However, the $\lambda_{mz}$ values are lower than the predictions by Connelly and Kokini [30], who studied a fully filled sigma blade mixer, which indicates that a partially filled mixer seems to have a decreasing effect on how well dispersed the particles will be in the suspension. Note that the presence of $\lambda_{mz}$ values between 0 and 0.1 is mainly due to the viscoplastic effect that makes the suspension more prone to have zones with a shear rate of zero and therefore limited mixing.

**Figure 10:**
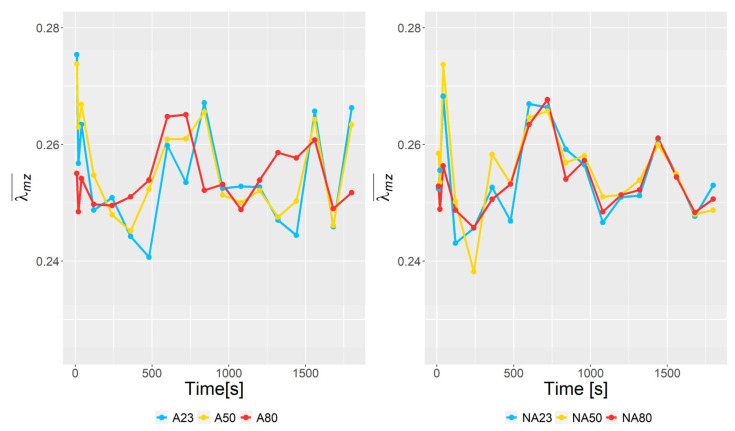
The mean value of $\lambda_{mz}$ for different time step values.

Figure 10 shows the mean value, $\bar{\lambda_{mz}}$, illustrated at different times. It is seen that the mean value fluctuates by ~10%. The fluctuations are most likely due to the free surface of the mixture (i.e., the mixer is partially filled).

### 3.5. Distributive Mixing

**Figure 11:**
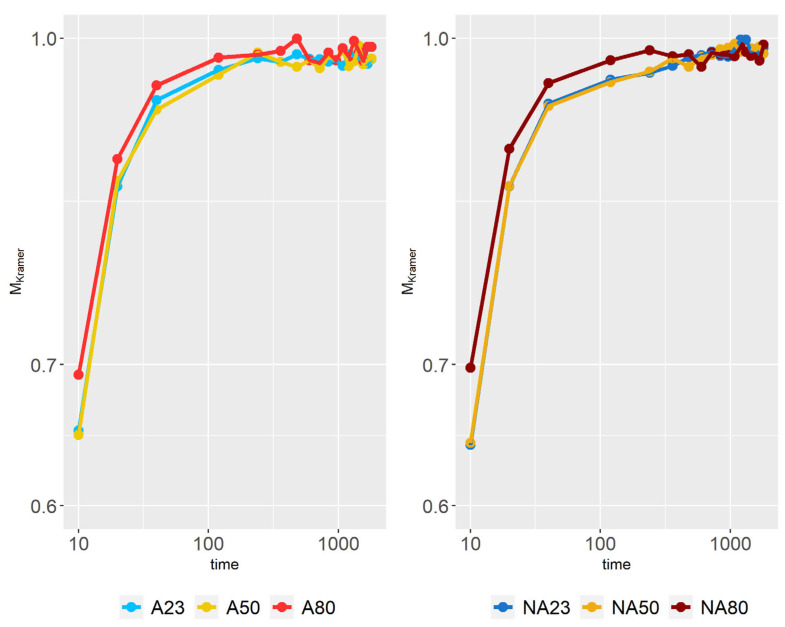
Kramer mixing index at different time values.

In Figure 11, the Kramer mixing index is illustrated at different time values for all simulations except *CA*23. The results show that all simulations obtain a high $M_{Kramer}$ value (i.e., close to 1) after 2 min, which indicates that the particles in the suspension end up being well distributed. *A*80 and *NA*80 reach a high $M_{Kramer}$ value the fastest (due to the lower viscosity at high temperatures), but this is marginally faster than the rest, thereby highlighting that energy potentially can be saved by not preheating the mixture without compromising the mixing of the suspension. Similarly, the results show that no substantial gain will be obtained regarding the mixing time by applying heat during the process. One should keep in mind that at a fixed mixing velocity, the Sigma blades require more energy at low temperatures due to the higher viscosity of the material. In addition, the Sigma blades will be exposed to larger forces at high viscosities, which can affect the blades’ lifespan.

## 4. Conclusions

This work presents a non-isothermal, non-Newtonian CFD model that simulates a partially filled Sigma blade mixer by taking into account viscous heating and the free surface of the suspension. The model is calibrated against physical temperature measurements, and good agreement between the experimental and numerical results is obtained. The results also highlight the importance of accounting for viscous heating when modeling the mixing of the suspension at hand. A numerical parameter study (i.e., varying wall temperature and pre-heating) was made in order to improve the understanding of the mixing conditions. The results of the model showed that the dispersive mixing index, $\bar{\lambda_{mz}}$, was primarily between 0 and 0.5, and it was not affected much by changing the wall and pre-heating temperature. Fluctuations of approximately 10% were found in the dispersive mixing index for most simulations, which was associated with the partially filled mixer (i.e., free surface of the suspension). This can be seen as a limitation of the mixing index. The model predicted via the Kramer mixing index that the suspension in all simulations would be well distributed after 1800 s. In addition, the results illustrated that pre-heating as well as heating during the process did not provide a substantial gain when it came to how fast a high Kramer index would be obtained. This illustrated that one could potentially save energy and time by eliminating the heating. This was a very interesting finding, as, intuitively, one could have expected that heating would lead to lower viscosity and therefore substantially faster mixing. In future work, focus will be put on extending the model to account for the effect of powder concentration variations on the viscosity and thermal conductivity, as this can especially affect the initial phase of the mixing.

## Figures and Tables

**Table 1:** Physical data for the fluid.

Name of the Property	Values	Unit
Density	1133	$\frac{\mathrm{kg}}{m^{3}}$
Heat capacity	1389	$\frac{J}{\mathrm{kg}\cdot {}^{\circ}C}$
Heat conductivity	$\begin{array}{c} k\left(t\leq 23\;{}^{\circ}C\right)=0.52\\ k\left(23\;{}^{\circ}C<t<71\;{}^{\circ}C\right)=-1.88\cdot 10^{-3}\;t+0.56\\ k\left(t\geq 71\;{}^{\circ}C\right)=0.43 \end{array}$	$\frac{J}{m\cdot K}$

**Table 2:** Viscosity data of the simulated fluid.

Symbol	Value	Unit
λ	1	s
n	−0.295	-
$\mu_{0}$	$4.35\cdot 10^{8}$	Pa·s
$\mu_{\infty }$	2.9	Pa·s
a	−20	-
b	−95.2	°C
$t^{ref}$	20	°C
$\gamma_{c}$	1	$s^{-1}$

**Table 3:** Information about the simulations.

Simulation ID	Boundary Condition	Initial Condition	Simulation Time
CA23	Adiabatic	23 °C	900 s
A23	Adiabatic	23 °C	1800 s
A50	Adiabatic	50 °C	1800 s
A80	Adiabatic	80 °C	1800 s
NA23	80 °C	23 °C	1800 s
NA50	80 °C	50 °C	1800 s
NA80	80 °C	80 °C	1800 s

## Data Availability

Data is available on request.
